# Epidemiological dynamics and molecular characteristics of HIV-1 among transgender women in the Central-West region of Brazil

**DOI:** 10.3389/fpubh.2025.1679535

**Published:** 2025-11-05

**Authors:** Bruno Vinícius Diniz e Silva, Sylvia Lopes Maia Teixeira, Paulie Marcelly Ribeiro dos Santos, Larissa Silva Magalhães, José Henrique Pilotto, Ingebourg Georg, Carlos Silva de Jesus, Rayana Katylin Mendes Da Silva, Karlla Antonieta Amorim Caetano, Robert Lewis Cook, Regina Maria Bringel Martins, Sheila Araujo Teles, Monick Lindenmeyer Guimarães, Megmar Aparecida dos Santos Carneiro

**Affiliations:** ^1^Institute of Tropical Pathology and Public Health, Federal University of Goiás, Goiânia, Goiás, Brazil; ^2^Laboratory of AIDS and Molecular Immunology, Oswaldo Cruz Institute, Rio de Janeiro, Brazil; ^3^Pontifical Catholic University of Goiás, Goiânia, Goiás, Brazil; ^4^Faculty of Nursing, Federal University of Goiás, Goiânia, Goiás, Brazil; ^5^Evandro Chagas National Institute of Infectious Diseases, Rio de Janeiro, Brazil; ^6^Department of Epidemiology, College of Public Health and Health Professions, and College of Medicine, University of Florida, Gainesville, FL, United States

**Keywords:** HIV-1, transgender women, epidemiology, genetic diversity, transmission network, sexual behavior

## Abstract

**Introduction:**

The human immunodeficiency virus (HIV) epidemic disproportionately affects key populations, including transgender women (TGW). Understanding the epidemiological and molecular characteristics on HIV-1 infection among TGW in Brazil, a continental country, is important to support decisions about public health policies. Therefore, this cross-sectional study aimed to evaluate the HIV prevalence and associated risk factors, recency of infection, genetic diversity of HIV-1, transmission clusters, and drug resistance mutations among TGW in Goiás, a state in the Central-West region of Brazil.

**Methods:**

A total of 440 participants from three cities in Goiás (Goiânia, Itumbiara, and Jataí) were recruited using Respondent-Driven Sampling. Serum samples were screened for anti-HIV antibodies using rapid tests and an enzyme-linked immunosorbent assay. Recent acquisitions were identified from plasma samples using the Limiting Antigen Avidity assay. Deoxyribonucleic acid was extracted from blood samples, and the partial polymerase gene (protease/reverse transcriptase region) was amplified using a nested polymerase chain reaction. Sequences were subtyped, analyzed for potential transmission networks, and assessed for drug resistance using the Stanford HIVdb program.

**Results:**

Most participants were young, single, self-identified as Black or mixed-race, and had a secondary education level or less. Many reported early sexual debut and multiple high-risk behaviors for sexually transmitted infections. The overall prevalence was 27.3%, with 43.2% classified as recent acquisitions and 56.8% as long-standing. Being over 25 years of age and engaging in chemsex were significantly associated with infection (*p* < 0.05). Regarding genetic diversity, subtype B was the most prevalent, followed by F1 and C. Ten transmission clusters were identified; each composed of at least two sequences. High prevalence of drug resistance mutations (39.1%) was observed among TGW.

**Conclusion:**

These findings underscore the high burden of HIV-1 among TGW in the Central-West region of Brazil and highlight the importance of serological and molecular surveillance in guiding targeted interventions aimed at preventing HIV acquisition and formulating well-informed public health policies for this key population.

## 1 Introduction

The World Health Organization (WHO) reported approximately 1.3 million new human immunodeficiency virus (HIV) infections worldwide in 2024, with 40.8 million people living with HIV (PLWHIV) (1). However, the HIV epidemic disproportionately affects key populations including men who have sex with men (MSM), transgender and gender-diverse individuals, sex workers, people who inject drugs, and people in prisons and other closed settings compared to the general population. Notably, the median HIV prevalence among transgender individuals was 9.2%, which is substantially higher than the global adult population estimate of 0.8% (ages 15–49) (2). Structural stigma and economic vulnerability experienced by transgender individuals increase their risk of HIV and other sexually transmitted infections (STIs) (3).

According to Spizzirri et al. (4), in Brazil, nearly three million people are estimated to be gender-diverse, representing approximately 2% of the adult population. Additionally, over 1 million Brazilians may identify as transgender. Despite these numbers, data on this population remain limited, especially in regions distant from the country’s HIV epicenter.

In Brazil, HIV epidemic is predominantly prevalent among those aged 15–49 years, with a national prevalence of 0.6% in 2023 (5). A biobehavioral survey conducted across 10 Brazilian cities reported HIV prevalence rates ranging from 22.5 to 71.5% among transgender women (TGW) (6). This population frequently reports risk behaviors for HIV acquisition, such as having multiple sexual partners and practicing chemsex or sexualized drug use (7). A study conducted from 2018 to 2020, using the Limiting Avidity assay as part of a recent infection testing algorithm, identified an alarmingly high HIV incidence among TGW in Rio de Janeiro (9.16%) (8).

This population presents ongoing challenges for public health stakeholders. Stigma and discrimination discourage TGW from accessing healthcare services (9), negatively affecting HIV testing, early diagnosis, treatment initiation, and adherence to antiretroviral therapy (ART) (10). Poor or non-adherence can lead to the development of HIV drug resistance over time (11).

Understanding the epidemiological dynamics and molecular characteristics of HIV infection among TGW is essential for formulating informed public health strategies and expanding access to HIV preventive interventions tailored to this population. Furthermore, to date, there is no available data regarding the HIV epidemic in the transgender population in Goiás, a state in the Central-West region of Brazil, which is geographically distant from the HIV epidemic epicenter in Brazil, a country with continental dimensions and with important differences in the dynamics of the HIV epidemic among geographic regions. Additionally, demographic and behavioral information regarding this population remain limited.

Herein, to fill these surveillance gaps and, therefore, to contribute to the knowledge about epidemiological and molecular data on HIV-1 infection among TGW in Brazil, we conducted this study aimed to evaluate HIV prevalence and associated risk factors, the frequency of recent HIV acquisitions, as well as HIV-1 genetic diversity, transmission clusters, and drug resistance mutations (DRM) among TGW in Goiás, Central-West region of Brazil.

## 2 Materials and methods

### 2.1 Study design and population

This cross-sectional study was conducted among TGW recruited between April 2018 and November 2019 in three cities: Goiânia, Itumbiara, and Jataí located in the state of Goiás, Central-West region of Brazil. These cities were selected because they either have specialized health facilities for TGW or have plans to implement such services. Additionally, they are recognized as key routes for prostitution and sex tourism in the region (12).

Eligible participants were individuals assigned male at birth who self-identified as female. The minimum required sample size was 454 TGW, calculated using a significance level of 95% (< 0.05), a 5.0% margin of precision, a design effect of 2.0, and an estimated HIV prevalence of 17.7% (13).

### 2.2 Study recruitment

Participant recruitment was conducted using the Respondent-Driven Sampling (RDS) method (14). Initially, formative research was carried out among TGW who held leadership roles in their communities. These individuals helped determine data collection sites, the form and value of incentives for participation, the expected time commitment, and the key TGW who would serve as initial recruiters (referred to as “seeds”). The RDS process began with a non-random selection of these seeds from within the target population. Each seed recruited three new participants, who then each recruited three more, and so on. Each participant received R$10 (approximately US$ 2.55) as a participation incentive, plus an additional R$10 for each successfully recruited peer who also participated in the study.

All participants were informed in advance about the study’s purpose, and those who agreed to participate signed a form indicating their free and informed consent. Trained staff conducted face-to-face interviews using a structured questionnaire to gather information on sociodemographic characteristics, sexual behaviors, drug use, prior HIV testing, and current use of ART. Following the interviews, blood samples were collected from all participants for laboratory testing.

### 2.3 Laboratory tests

#### 2.3.1 Detection of anti-HIV antibodies

All participants were tested for anti-HIV-1/2 antibodies using rapid diagnostic tests (Immunochromatography: Bioclin HIV Tri line, Quibasa, Belo Horizonte, Brazil; Rapid test: R DPP® HIV 1/2, Bio-Manguinhos, Rio de Janeiro, Brazil). We also tested serum samples using an enzyme-linked immunosorbent assay (ELISA; Genscreen™ ULTRA HIV Ag-Ab, Bio-Rad, Marnes-la-Coquette, France). Samples that tested positive on both the rapid tests and ELISA were considered anti-HIV positive.

#### 2.3.2 HIV recency testing

Recent HIV acquisitions were identified from available stored plasma samples using the Maxim HIV-1 Limiting Antigen Avidity assay (LAg-Avidity EIA; Maxim Biomedical, USA). To prevent the overestimation of long-standing acquisitions, individuals who reported using ART were excluded from recency testing. Samples with a spectrophotometer read below or equal to a 1.5 threshold were classified as recent acquisitions. A mean duration of recent acquisition was assumed to be 161 days (95% confidence interval [95% CI]: 148–174)^1^.

### 2.4 Genetic diversity analysis

#### 2.4.1 DNA extraction, amplification, and sequencing

HIV-1 DNA was extracted from blood samples using the QIAamp DNA Blood Mini Kit (Qiagen, Hilden, Germany) for use in a nested polymerase chain reaction (PCR) targeting the *pol* gene region. The nested PCR amplified a 1,478 bp fragment (positions 2077–3,574 relative to HXB2) covering the protease and partial reverse transcriptase regions. Amplicons were purified and sequenced as previously described (15, 16).

Sequence editing was performed using the SeqMan module of DNASTAR 4.0 software, and alignments were carried out with pure subtype references and circulating recombinant forms (BF, BC, and CF) from the Los Alamos HIV Sequence Database using the Clustal W program implemented in MEGA 11.0 software (17). The final *pol* alignment covered a 1,302 bp fragment, corresponding to nucleotides 2,253 to 3,554 of the HXB2 genome. Phylogenetic analysis was performed using the neighbor-joining (NJ) method and the Tamura-Nei substitution model in MEGA 11.0. Bootstra*p* values > 90% (1,000 replicates) were considered statistically significant. Potential recombinant sequences were identified using a bootscan analysis in the SimPlot version 3.5.1 program (sliding window of 200 bp, step size of 10 bp). All nucleotide sequences have been deposited in the GenBank database under accession numbers V794413 to PV794494.

#### 2.4.2 Transmission cluster analysis

Sequences that clustered together with high bootstrap support (> 0.90) in the NJ tree were analyzed to identify potential transmission clusters. To this end, we used the nucleotide Basic Local Alignment Search Tool (BLASTn) (18) to retrieve reference sequences with high similarity (> 95%). Before performing phylogenetic analyses to confirm transmission clusters according to HIV-1 subtypes, DRM positions were stripped from each alignment, resulting in a 1,261 bp fragment spanning nucleotides 2,253 to 3,514 of the HXB2 genome. Sequences were considered part of a transmission cluster if they branched together and met the criteria of an aLRT (> 90) and a mean pairwise genetic distance (≤ 4.5%).

#### 2.4.3 Drug resistance mutation analysis

We submitted the sequences to the Stanford HIV Database (https://hivdb.stanford.edu/page/algorithm-updates/) for DRM identification.

### 2.5 Data analysis

Prevalence and 95% CIs were estimated using the Respondent-Driven Sampling Analysis Tool (RDSAT) version 7.1.46. RDSAT provided weights for each participant based on their social network size, and analyzed homophily, network size, recruitment waves, and equilibrium. To measure network size, we asked each participant: “How many transgender women do you know personally?” To correct for outliers (19), network sizes were constrained to the interval of 3 and 150. Values less than three or greater than 150 were replaced by 3 and 150, respectively (20). Data and weights generated by RDSAT were exported to STATA v.17 for modeling. We used RDSAT with 15,000 bootstraps. Bivariate logistic regression analysis was performed to identify variables associated with the outcome (anti-HIV positive). The multivariate logistic regression model included variables with *p*-values < 0.25 from the bivariate analysis (21). A p-value < 0.05 was deemed statistically significant. We assessed multicollinearity using variance inflation factors and tolerance; all retained variables met acceptable thresholds. Recruitment networks, including the distribution of HIV-1 cases, were visualized with NetDraw software (22).

### 2.6 Ethical statement

The Federal University of Goiás Ethics Committee for Human Research approved the study under the protocol CAAE 77481417.5.0000.5083 and 2.358.818.

## 3 Results

Eight seeds (five in Goiânia, one in Jataí, and two in Itumbiara) distributed 1,312 invitations, resulting in 440 individuals agreeing to participate in the study: 285 from Goiânia, 74 from Itumbiara, and 81 from Jataí. This total represents 96.9% of the calculated minimum required sample size (*N* = 454). The number of participants recruited per seed ranged from 2 to 141, and recruitment was completed over 64 weeks through 1 to 14 waves (Supplementary Table S1).

Table 1 shows both crude and RDSAT-weighted prevalence estimates of participant characteristics. Based on weighted data, 56.2% of participants were aged 25 years or younger, the majority were single (81.7%), and 72.6% identified as Black or mixed race. Only 18.3% had more than a secondary education, and 13.6% reported a monthly income of BRL 2,000 (Brazilian *reais*) or more. Among TGW, 46.9% had their first sexual experience at age 13 or younger, and 39.7% reported having experienced sexual assault. A total of 30.8% identified as sex workers. Nearly all participants (97.1%) reported engaging in receptive anal sex, and 55.7% also reported insertive anal sex. Most participants (61.7%) reported inconsistent condom use during anal sex, 64.4% reported engaging in group sex, and 77.5% had more than ten sexual partners in the past week. Chemsex was reported by 60.0% of participants. Regarding sexual partners, 75.3% had sex exclusively with men, 15.1% with men and women, and 16.5% with TGW. A previous STI diagnosis was reported by 39.8%. Non-injection illicit drug use was reported by 60.2%, while declared injection drug use was reported by only 1.7%.

**Table 1:** Crude and RDS-weighted characteristics of transgender women in Goiás, Brazil, 2018–2019.

Variable	n	% Crude	% RDS*	95% CI
Age (median: 25 years; IQR:9)				
≤ 25	245	55.7	56.2	47.2–64.8
> 25	195	44.3	43.8	35.2–52.8
Schooling (median 11; IQR: 3)				
More than secondary level	202	10.9	18.3	11.2–25.9
Until secondary level		61.1	56.5	48.4–65.4
Until elementary level	238	28.0	25.2	17.9–32.2
Monthly income (BRL; median: 2,000; IQR: 2,000)^a^				
>2,000	104	23.6	13.6	8.3–19.8
1,000–2,000	211	48.0	45.6	37.8–53.9
<=1,000	125	28.4	40.8	32.1–49.4
Marital status				
Single	372	84.7	81.7	72.6–87.8
Married/consensual union	64	14.6	18.2	12.1–27.3
Divorced//widow	3	0.7	0.1	0.0–0.3
No information: 1				
Skin color/race (self-defined)				
White	90	20.6	20.3	13.8–26.9
Black or mixed race	312	71.2	72.6	65.3–79.8
Other	36	8.2	7.2	3.9–11.3
No information: 2				
First sex (age; median: 13; IQR: 4)				
>13	195	44.3	53.1	45.0–61.9
≤13	245	55.7	46.9	38.1–55.0
Ever sex assault				
No	227	51.8	60.3	52.4–69.0
Yes	211	48.2	39.7	31.0–47.7
No information: 2				
Sex worker				
No	182	41.4	69.2	58.5–78.2
Yes	258	58.6	30.8	21.8–41.5
Receptive anal sex				
No	11	2.5	2.9	4.0–6.4
Yes	428	97.5	97.1	93.6–99.6
No information: 1				
Insertive anal sex				
No	113	25.7	44.3	35.3–53.4
Yes	326	74.3	55.7	46.6–64.7
No information: 1				
Consistent condom uses in anal sex				
Yes	156	36.2	38.3	28.8–45.2
No	275	63.8	61.7	54.8–71.2
No information: 9				
Number of sexual partners (last 7 days; median: 10; IQR:29)				
<=1	83	18.9	9.2	5.2–14.2
2–10	108	24.5	13.3	8.3–19.1
>10	249	56.6	77.5	69.5–84.6
Group sex				
No	86	19.6	35.6	25.9–45.7
Yes	353	80.4	64.4	54.3–74.1
No information: 1				
Chemsex				
No	107	24.5	40.0	30.8–49.1
Yes	330	75.5	60.0	51.0–69.2
No information: 3				
Sex with man only				
No	188	43.1	24.7	18.3–32.1
Yes	248	56.9	75.3	67.9–81.7
No information: 4				
Sex with man and woman				
No	297	68.1	84.9	79.3–89.2
Yes	139	31.9	15.1	10.8–20.7
No information: 4				
Sex with transgender woman				
No	322	73.9	83.5	76.7–89.2
Yes	114	26.1	16.5	10.8–23.4
No information: 4				
Previous STI				
No	221	50.2	60.2	51.8–68.3
Yes	219	49.8	39.8	31.7–48.2
Non-injection illicit drug use				
No	114	26.0	39.8	30.9–49.4
Yes	325	74.0	60.2	50.6–69.1
No information: 1				
Injection illicit drug use				
No	425	97.3	98.3	96.0–99.8
Yes	12	2.7	1.7	0.2–4.0
No information: 3				

RDS*: Respondent Driven Sampling; CI: confidence interval; IQR: interquartile range; BRL: Brazilian currency (reais); STI: sexually transmitted infection. ^a^BRL 3.91 was equivalent to USD 1 during the study period.

**Figure 1:**
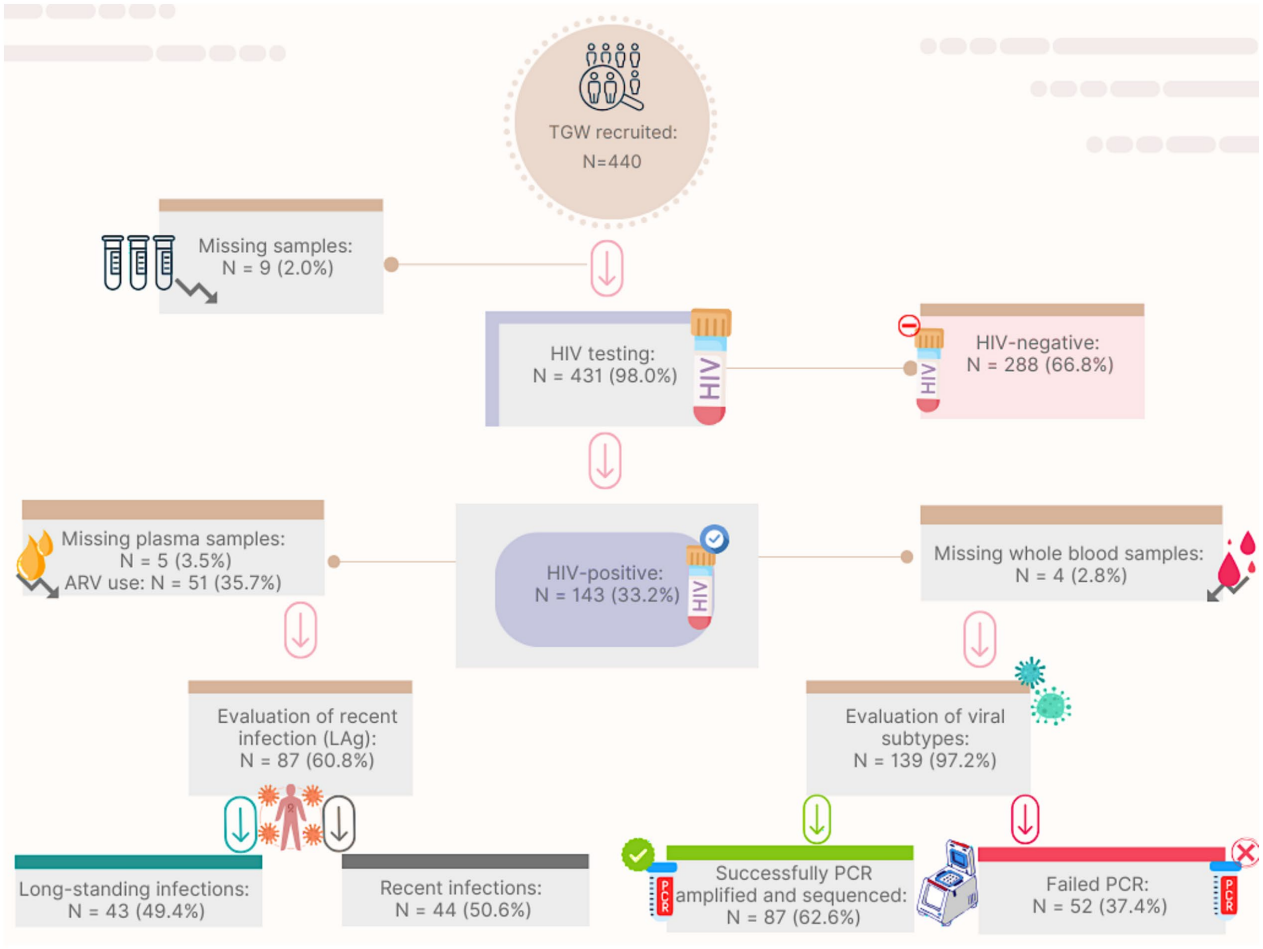
Flow-chart for HIV recent acquisition screening and genetic diversity analysis among transgender women from Goiás, Brazil. TGW: transgender women; HIV: Human Immunodeficiency Virus; ARV: antiretroviral; LAg: limiting-antigen assay; PCR: polymerase chain reaction.

A total of 440 TGW were tested for anti-HIV-1/2 using rapid tests, and 431 were also tested using ELISA. Nine of these did not have samples available for ELISA. Among those tested, 143 out of 431 (33.2%) had positive results on at least two anti-HIV assays and were therefore considered HIV positive. Of the 143 individuals who tested positive for HIV, 51 reported current ARV use and were excluded from the recency analysis. Plasma samples were available for 87 out of the remaining 92 participants (94.6%) for LAg testing. Of these, 44 (50.6%) were classified as recent HIV acquisitions, and 43 (49.4%) were long-standing cases (Figure 1). There were no statistically significant sociodemographic differences between TGW with recent and long-term acquisitions (*p* > 0.05).

The RDSAT-weighted prevalence of HIV was 27.3% (95% CI: 20.8–35.4). Based on LAg avidity testing, the weighted prevalence of recent and long-term HIV acquisition was 43.2% (95% CI: 14–58) and 56.8% (95% CI: 42.1–86), respectively.

Of 440 TGW, 385 (87.5%) reported previous HIV testing. Among them, 77/385 (20%) reported a positive HIV serostatus, and 51/77 (66.2%) confirmed ART use. Furthermore, among 297 participants who self-reported as HIV-negative, 52 (17.5%) tested positive in this study, and 34 of the 49 with LAg results (69.4%) were classified as recent infections.

Table 2 shows the variables associated with HIV infection. In the bivariate analysis, age and chemsex were significantly associated with HIV (*p* < 0.05). These variables, along with others with *p*-values < 0.25 (insertive anal sex, group sex, previous STI, and sex with TGW), were included in the multivariate logistic regression model and remained independently associated with HIV infection. TGW aged 25 years or older had 2.83 times greater odds of being HIV positive (95% CI: 1.31–6.15) compared to younger participants. Those who reported chemsex had 2.65 greater odds of being infected with HIV (95% CI: 1.14–6.13) than those who did not report this behavior.

**Table 2:** Bivariate and multivariate analysis of variables associated with HIV among transgender women in Goiás, Brazil, 2018–2019.

Variable	*p* value	Bivariate (RDS weighted)	*p* value	Multivariate (RDS weighted)
		OR (95% CI)		Adjusted OR (95% CI)^a^
Aged >25 years	0.021	2.34 (1.14–4.83)	**0.009**	**2.83 (1.31–6.15)**
Scholarly^b^				
More than secondary level		1.00		
Until secondary level	0.793	0.86 (0.28–2.61)		
Until elementary level	0.959	1.03 (0.31–3.42)		
Monthly income (BRL)^c^				
≥ 2,000		1.00		
1,001–1,999	0.987	0.99 (3.40–2.48)		
≤ 1,000	0.597	0.72 (0.28–2.08)		
Non-White	0.693	0.831 (0.33–2.08)		
Single/divorced/widow	0.531	0.73 (0.27–1.97)		
First sex at 13 years old or less	0.508	0.79 (0.39–1.60)		
Being sex worker	0.936	0.97 (0.50–191)		
Insertive anal sex	0.138	1.79 (0.83–3.88)	0.439	1.40 (0.60–3.25)
Receptive anal sex	0.274	2.91 (0.43–19.78)		
Inconsistent condom use in anal sex	0.965	1.02 (0.47–2.20)		
Ever suffered coercive sex	0.830	0.93 (0.45–1.89)		
Number of sexual partners (last 7 days)				
< 2		1.00		
2–10	0.815	0.87 (0.28–2.74)		
>10	0.626	0.78 (0.29–2.12)		
Group sex	0.098	2.16 (0.87–5.35)	0.761	1.167 (0.433.17)
Chemsex	0.049	2.27 (1.00–5.12)	**0.023**	**2.65 (1.14–6.13)**
Previous STI	0.065	1.98 (0.96–5.483)	0.141	1.74 (0.83–3.65)
Sex with man only	0.391	0.72 (0.34–1.53)		
Sex with man and woman	0.333	1.52 (0.65–3.53)		
Sex with TGW	0.235	1.70 (0.71–4.10)	0.659	1.24 (0.47–3.28)
Non-injection illicit drug use	0.672	1.18 (0.55–2.51)		
Injection illicit drug use	0.405	0.40 (0.048–3.42)		

RDS: Respondent Driven Sampling; OR: Odds Ratio; CI: confidence interval; BRL: Brazilian currency (reais); STI: sexually transmitted infection; TGW: transgender woman.

^a^Adjusted by age, insertive anal sex, group sex, chemsex, previous STI and sex with transgender woman.

^b^Scholarly: elementary level ≤ 9 years of formal education, secondary level = 10 to 12 years of formal education; ^c^BRL 3.91 was equivalent to USD 1 during the study period.*p* values < 0.05 were considered statistically significant.

Of the 143 TGW living with HIV, 139 had blood samples available for DNA extraction. Among these, 87 samples were successfully PCR amplified and sequenced (63%, Figure 1). Phylogenetic analyses revealed that HIV-1 subtype B was the most prevalent clade (55.2%) in our sampling, followed by F1 (17.2%), C (15%), and recombinant forms (12.6%). The recombinants included 9.2% BF (one CRF39 and seven URFs), 2.3% BC, and 1.1% CF (Supplementary Figure S1).

**Figure 2:**
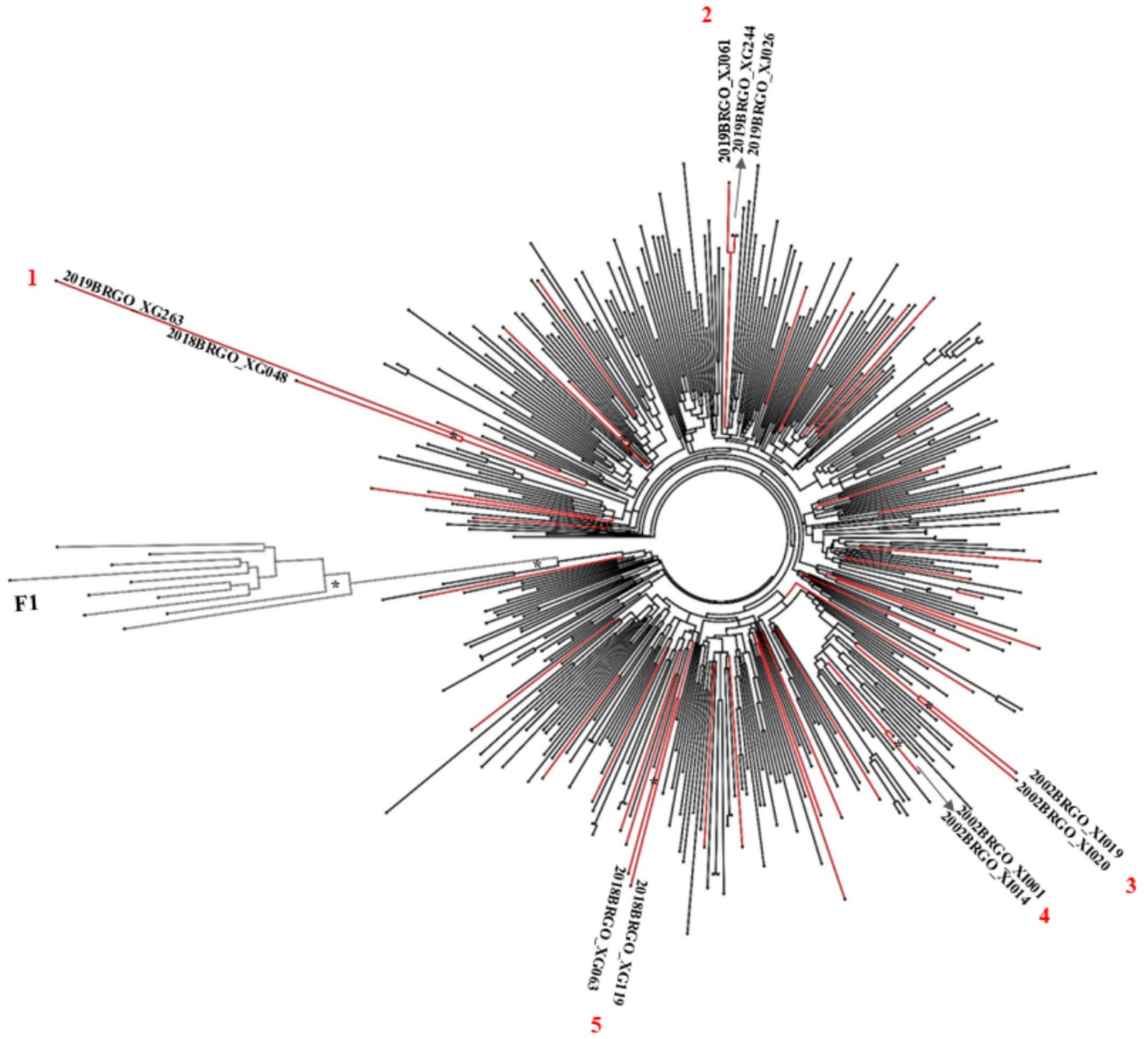
ML phylogenetic tree of 45 HIV-1 subtype B sequences in PR/RT from transgender women in Goiás. The analyzed PR/RT alignment covered a fragment of 1,261 bp, corresponding to nucleotides 2,253 to 3,514 relative to HXB2 genome, drug-resistance mutation positions were stripped from each alignment. Reference sequences retrieved from GenBank are not labeled. ALRT values are represented only if greater > 0.90 with an asterisk. Possible HIV-1 transmission clusters were indicated by numbers. Three short sequences were not included in the analyses.

**Figure 3:**
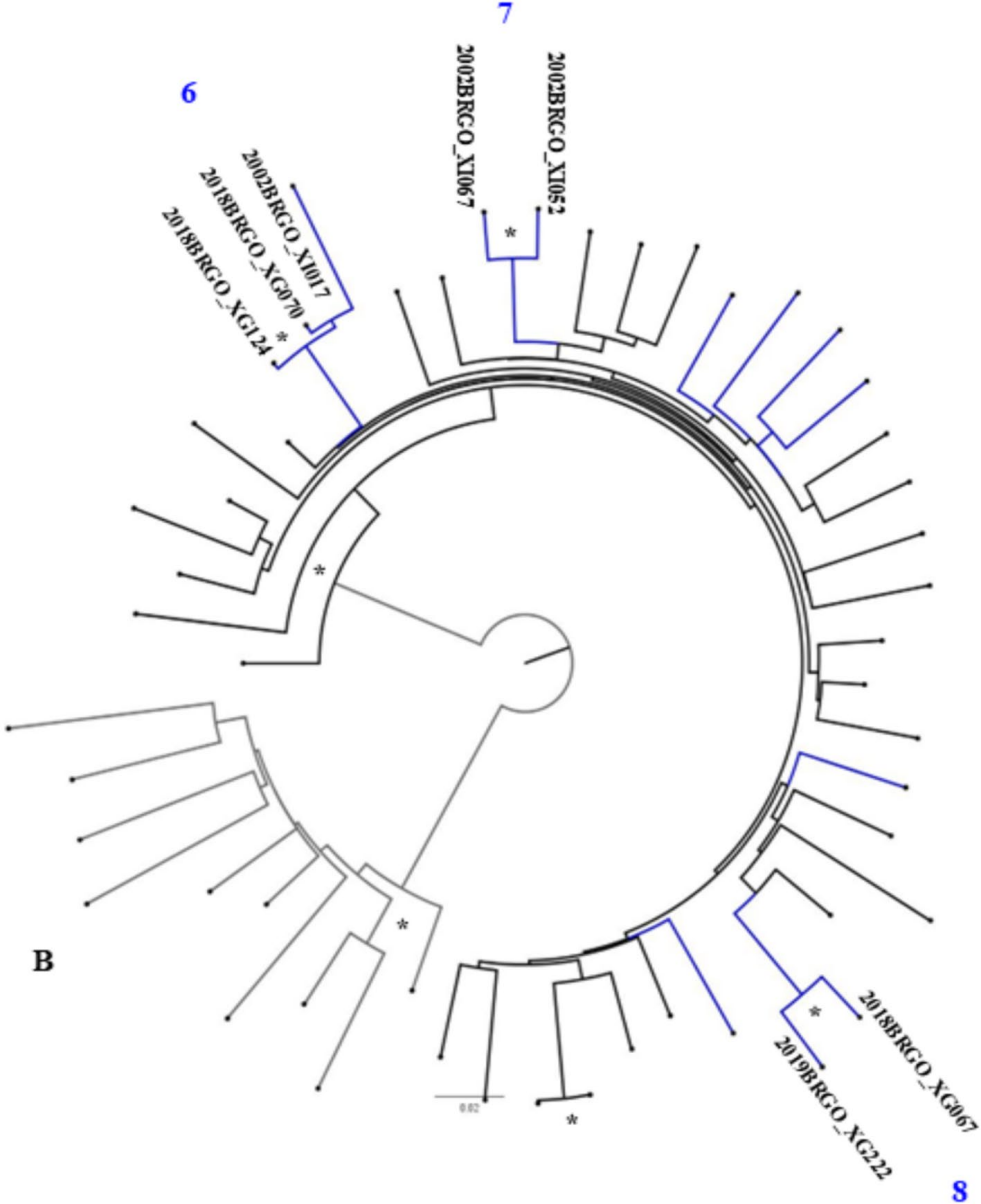
ML phylogenetic tree of 13 HIV-1 subtype C sequences in PR/RT from transgender women in Goiás. The analyzed PR/RT alignment covered a fragment of 1,261 bp, corresponding to nucleotides 2,254 to 3,514 relative to HXB2 genome, drug-resistance mutation positions were stripped from each alignment. Reference sequences retrieved from GenBank are not labeled. ALRT values are represented only if greater > 0.90 with an asterisk. Possible HIV-1 transmission clusters were indicated by numbers. Two short sequences were not included in the analyses.

**Figure 4:**
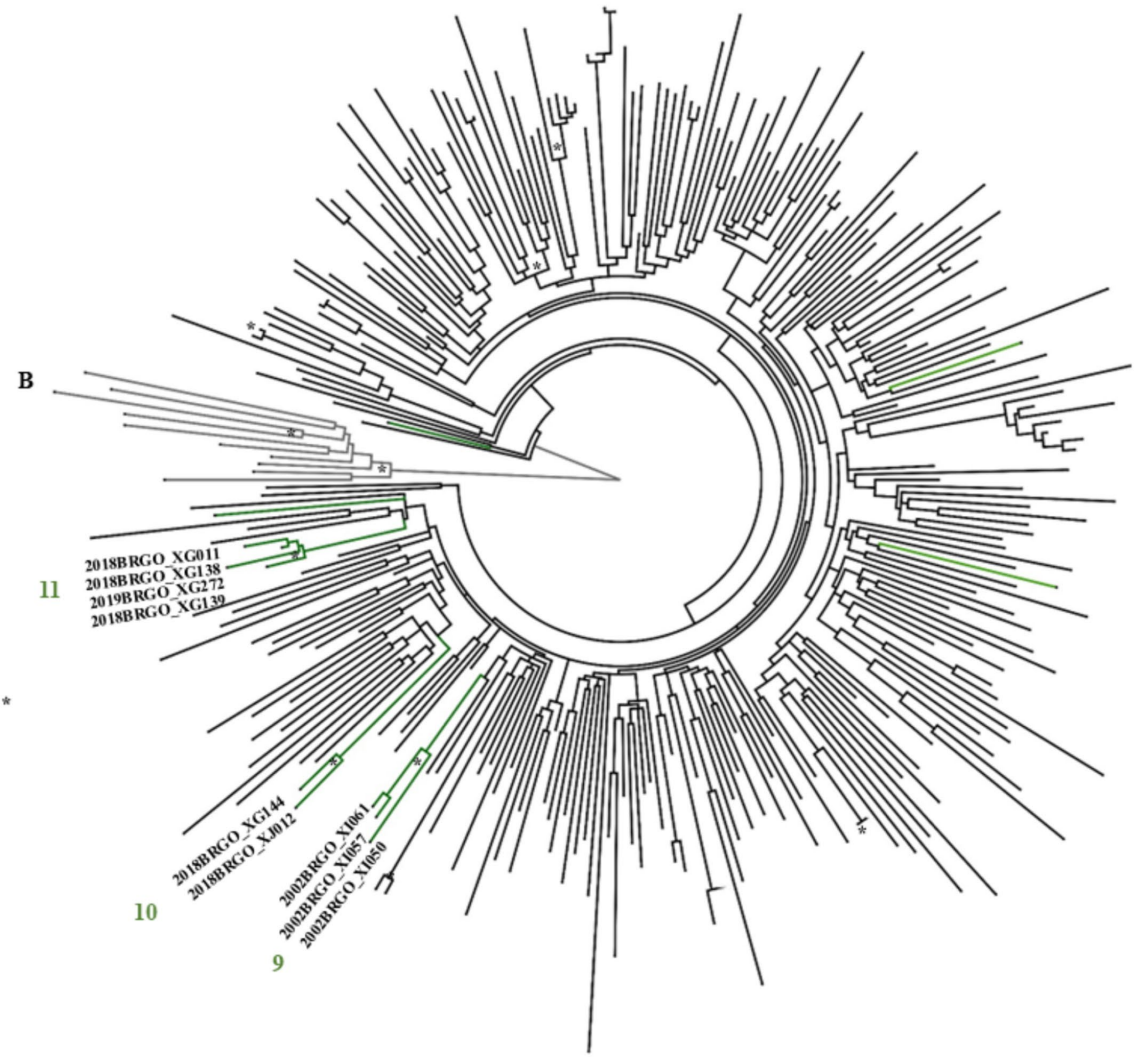
ML phylogenetic tree of 13 HIV-1 sub-subtype F1 sequences in PR/RT from transgender women in Goiás. The analyzed PR/RT alignment covered a fragment of 1,261 bp, corresponding to nucleotides 2,254 to 3,514 relative to HXB2 genome, drug-resistance mutation positions were stripped from each alignment. Reference sequences retrieved from GenBank are not labeled. ALRT values are represented only if greater > 0.90 with an asterisk. Possible HIV-1 transmission clusters were indicated by numbers.

**Figure 5:**
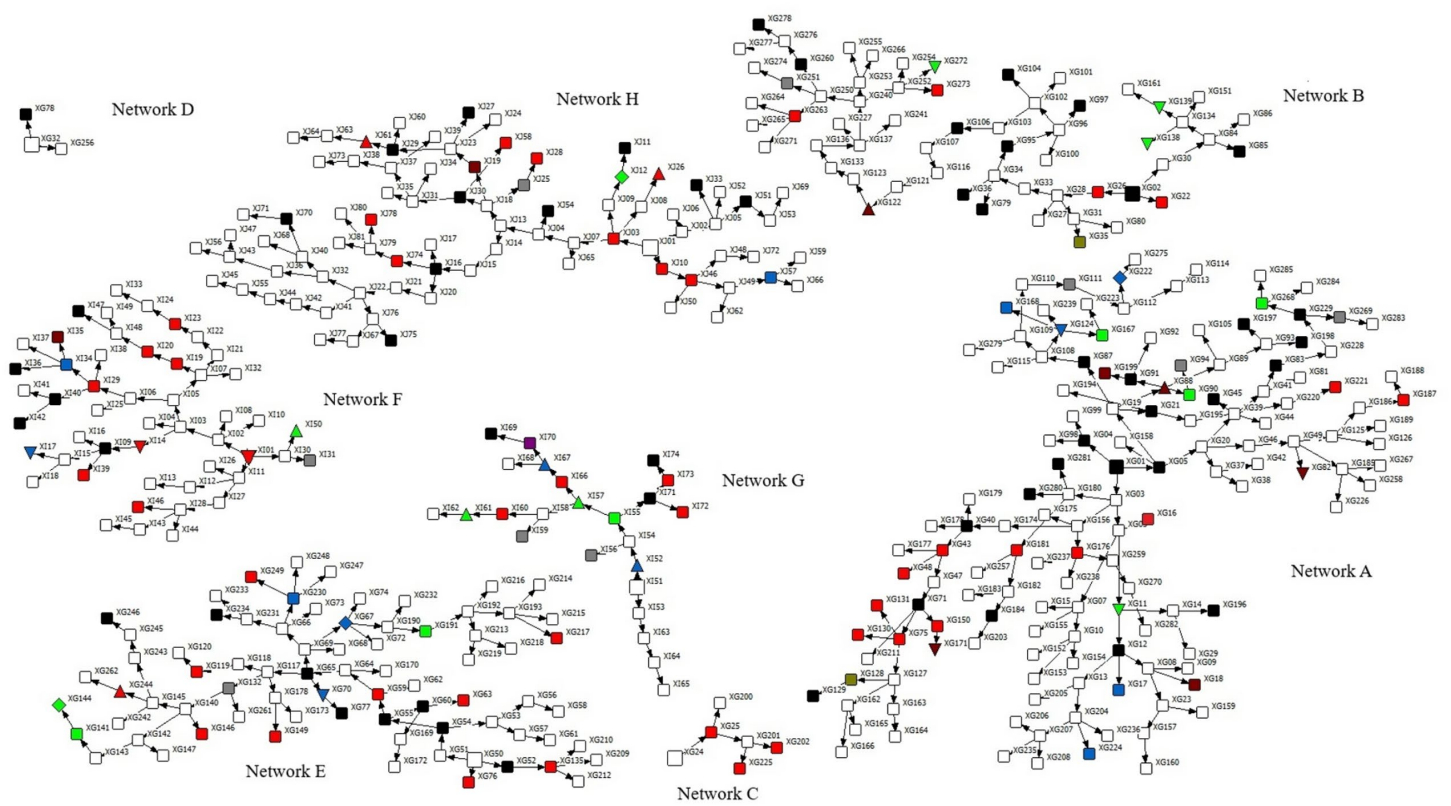
Recruitment networks (A–H) of 440 transgender women (TGW) in Goiás, Central-Western Brazil. Squared round represents each participant and their respective ID. Prefix XG define TGW recruited in Goiânia city, XI in Itumbiara city and XJ in Jataí city. The seeds are represented by larger symbols. HIV-1 seropositive TGW whose subtype was not identified are colored in black, blue (subtype C), green (subtype F), red (subtype B), brown (recombinant BF1), olive green (BC) and fuchsia (FC). Gray color defines undetermined samples. Clusters of subtype B are identified as uptriangle (cluster 2) and downtriangle (cluster 4). Clusters of subtype C are identified as downtriangle (cluster 6), uptriangle (cluster 7) and diamond (cluster 8). Clusters of subtype F are identified as uptriangle (cluster 9), diamond (cluster 10) and downtriangle (cluster 11). Cluster of recombinant BF1are identified as uptriangle (cluster 12) and downtriangle (cluster 13).

Maximum likelihood (ML) phylogenetic analysis (Figures 2–4) showed that 35.6% (31/87) of the sequences were grouped into 13 clusters, each comprising two to four sequences with aLRT > 0.90. Combined phylogenetic and genetic distance analysis (≤ 4.5%) confirmed transmission relationships in 10 of the 13 clusters: two were subtype B (clusters 2 and 4), three were subtype C (clusters 6 to 8), three were subtype F1 (clusters 9 to 11), and two were BF recombinants (clusters 12 and 13). Clusters 1, 3, and 5, all subtype B, did not meet the criteria to confirm a transmission cluster since they presented a mean pairwise genetic distance > 4.5% (Figures 2–4; Supplementary Table S2). We observed that sequences from three transmission clusters (4, 7, and 13) originated from TGW recruited in the same city and within the same recruitment networks (F, G, and A, respectively). In contrast, the remaining clusters (2, 6, and 8–12) included sequences from TGW recruited from different RDS networks and in the same or different cities (Figure 5), highlighting the TGW mobility among the studied cities and inter-network mixing.

DRMs were detected in 34 of the 87 (39.1%) subtyped sequences, some of them reflecting transmitted drug resistance mutations. Additionally, 44.8% (39/87) of individuals reported prior ARV use. Mutations associated with protease inhibitors (PI) were detected in six individuals (6.9%). Mutations linked to nucleoside reverse transcriptase inhibitors (NRTI) were found in 20 samples (23%), and non-nucleoside reverse transcriptase inhibitors (NNRTI) mutations were identified in 22 samples (25.3%). The most prevalent mutations were S68G (10/87, 11.5%) and M184V/I (7/87, 8%) for NRTIs, and K103N and V179D/E/T (each in 6/87, 6.9%) for NNRTIs (Supplementary Table S3).

## 4 Discussion

This study provides relevant epidemiological and molecular data on HIV-1 infection among TGW in Goiás, Central-West region of Brazil. Our findings reveal alarmingly high HIV burden in this population. To the best of our knowledge, only one previous study has examined the molecular epidemiology of HIV infection among TGW in this region. Conducted 10 years ago in Campo Grande City, Mato Grosso do Sul, that study used a convenience sample of 152 TGW—nearly three times smaller than ours—and reported molecular characteristics for only 11 sequenced HIV samples (23).

The sociodemographic characteristics of the TGW in our study are consistent with findings from other Brazilian studies and international studies involving this population (24–29). Most participants were young, self-identified as Black or mixed race, single, had less than a secondary level of formal education, and reported a monthly income below 2,000 BRL.

In Goiás, the HIV prevalence among TGW was 68 times higher (27.3%; 95% CI: 20.8–35.4) than that reported among Brazilian women aged 15 to 49 years (0.4%) (5). This rate is comparable to those observed among TGW in São Paulo (38%; 95% CI: 30.9–45.6) (30) and Rio de Janeiro (31.2%; 95% CI: 18.8–43.6) (31), cities in the Southeast region that bear a higher burden of HIV in Brazil (32). These findings underscore the elevated risk of HIV among TGW, even in regions far from the epidemic’s epicenter. A recent global systematic review and meta-analysis estimated a 19.9% HIV prevalence (95% CI: 14.7–25.1) among TGW (33).

Most TGW in our study (385/440, 87.5%) reported having been previously tested for HIV. Among them, 20% (77/385) reported being HIV positive, and 66.2% (51/77) of these individuals confirmed ART use. When the 52 newly diagnosed cases identified in this study are included, the proportion of TGW on ART drops to 39.5% (51/129), which falls far short of the Joint United Nations Programs on HIV/AIDS (UNAIDS) 95–95-95 targets: 95% of PLWHIV should know their status; 95% should receive ART; and 95% of those on ART should achieve viral suppression (1). These findings suggest that TGW in Goiás remain significantly underserved in the HIV care continuum and are far from reaching these global targets.

The high proportion of recent acquisitions found in the studied population (50.6%) highlight an elevated transmission rate and ongoing viral dissemination among TGW in Goiás. This finding, along with the high HIV prevalence and the gaps in treatment coverage, suggests that the current HIV prevention and treatment policies in Brazil are having a limited impact on this key population. These findings reinforce the urgent need for greater public investments to address the social determinants—such as low education attainment, poverty, and exposure to violence—that shape the lives of TGW and may contribute to risk-taking behaviors.

Similarly, a high frequency of risky behaviors has been reported among TGW in the US (34), and in the present study, most of these behaviors were associated with HIV infection in bivariate analysis. However, in the multivariate analysis, only older age and chemsex remained independently associated with HIV. The link between age and HIV infection is well documented and likely reflects cumulative exposure to risk over time (35, 36). Additionally, the perception of HIV risk may diminish with age, potentially leading to behaviors that increase vulnerability to infection. It is also notable that many TGW initiate sexual activity at a very young age, suggesting that HIV acquisition can occur as early as adolescence or early adulthood (37). Chemsex is frequently reported among TGW (7). According to Kcomt et al. (38), transphobia often drives both substance abuse and engagement in sex work. Substance abuse may serve as a coping mechanism to blunt emotional pain or trauma, and it can also be client-driven, either to enhance sexual arousal or as a requirement during encounters (39, 40). Moreover, Incera-Fernández et al. (41) reported in their systematic review that intervention practices are beneficial in addressing chemsex, including harm reduction programs and behavioral activation therapy, which have proven significantly effective in reducing risky behaviors, decreasing the frequency of chemsex participation, and improving the physical, mental, and sexual health of people with a history of chemsex. Therefore, the inclusion of TGW in harm reduction programs aimed at reducing chemsex can contribute to an improved quality of life and lower rates of HIV and other STIs.

In terms of HIV-1 subtypes, these results underscore the ongoing diversification of HIV subtypes within the TGW population. Also, they corroborate previous data from studies focused specific groups such as pregnant women and MSM in Goiás and a national study in HIV1 molecular diversity in Brazil (2008–2017), which reported an increasing trend in the prevalence of subtypes C and F1, alongside a gradual decline in subtype B (42).

Another important finding was that 28.7% (25/87) of the HIV-1 sequences were linked to at least one other sequence, forming 10 transmission clusters. Six sequences grouped within three clusters (4, 7, and 13) (Supplementary Table S2) originated from TGW and belonged to the same recruitment networks (F, G, and A, respectively) (Figure 5). These participants reported sex-related risk factors, such as unprotected sexual intercourse, group sex, and a history of STIs. In contrast, sequences from the remaining seven clusters (2, 6, and 8 to 12) were obtained from TGW affiliated with different recruitment networks. This pattern highlights the high geographic mobility and sexually interconnected networks of TGW spreading HIV and other STIs across cities.

The frequency of HIV-1 DRMs detected in this study (39.1%) was higher than that observed in two previous studies involving MSM—17.3% in Goiás and 12% in Mato Grosso do Sul (43, 44). Notably, 44.8% (39/87) of TGW included in our analysis reported being on ART. However, considering the first-line regimen recommended for PLWHIV in Brazil since 2024 (lamivudine/tenofovir+dolutegravir), approximately 30% of participants would be at risk of reduced treatment effectiveness due to the presence of resistance mutations. Thus, we highlight the timely diagnosis, the early initiation of antiretroviral therapy, and the retention of TGW in the healthcare system as key monitoring gaps.

A key strength of this study is the successful use of the RDS methodology, which enabled the recruitment of a large number of TGW and the generation of both epidemiological and molecular HIV data from a population in a region far away—such as the Central-West region of Brazil—that lies outside the usual focus of national HIV surveillance. Nonetheless, our study has some limitations. First, because all interviews were conducted face-to-face, some responses, particularly those related to sexual behaviors, may be subject to reporting bias. However, to minimize potential bias, measures such as the use of well-trained interviewers and private interview settings were implemented. The interaction between participants and well-trained interviewers is particularly important when working with hard-to-reach populations, such as TGW, since it plays a key role in fostering trust and rapport between interviewers and participants. Furthermore, RDS assumes participants invite people from their own network of relationships, which can contribute to the overrepresentation of some profiles and the underrepresentation of others. Despite these limitations, RDS is commonly used and is likely to continue being used for obtaining epidemiological data from hard-to-reach populations until more effective methods become available. Second, we were unable to apply the full recent infection testing algorithm, which includes CD4 + lymphocyte counts and HIV RNA quantification (viral load), due to limited access to these clinical data from public health databases. For this reason, our recent acquisitions’ estimates can be overestimated. Third, the low PCR amplification rate among HIV-positive samples may have affected the robustness of our molecular analyses, potentially leading to an underestimation of HIV subtypes and drug resistance mutations. Nevertheless, neither HIV-1 subtype distribution nor drug resistance mutations appear to have been biased, as they were consistent with previous studies (40, 43, 45). Despite these limitations, the study offers valuable insights into the epidemiology and molecular characteristics of HIV-1 infection in Brazilian TGW—a pivotal population that warrants greater inclusion in targeted HIV prevention strategies.

Our findings underscore the considerable impact of the HIV epidemic on TGW in the Central-West region of Brazil despite the implementation of public health prevention focused on key populations. There is an urgent need to develop more effective, inclusive policies and expand targeted diagnosis, interventions, and care for TGW. This includes access to pre-exposure prophylaxis (PrEP) for TGW to meet the specific needs of this vulnerable group, as well as expand HIV testing, strengthen HIV molecular surveillance, improve ART coverage, and address structural barriers faced by them, to curb the spread of HIV in Brazil.

## Data Availability

The datasets presented in this study can be found in online repositories. The names of the repository/repositories and accession number(s) can be found in the article/Supplementary material.

## Footnotes

^1^ https://www.maximbio.com

## References

[1] World Health Organization (WHO). HIV and AIDS [internet]. (2025). Available online at: https://www.who.int/news-room/fact-sheets/detail/hiv-aids

[2] UNAIDS. Global HIV & AIDS statistics — fact sheet [internet]. (2024) Available online at: https://www.unaids.org/en/resources/fact-sheet

[3] Mujugira A Kasiita V Bagaya M Nakyanzi A Bambia F Nampewo O. "you are not a man": a multi-method study of trans stigma and risk of HIV and sexually transmitted infections among trans men in Uganda. J Int AIDS Soc. (2021) 24:e25860. doi: 10.1002/jia2.25860, PMID: 34965322 PMC8716065

[4] Spizzirri G Eufrasio R Lima MCP de Carvalho Nunes HR Kreukels BPC Steensma TD. Proportion of people identified as transgender and non-binary gender in Brazil. Sci Rep. (2021) 11:2240. doi: 10.1038/s41598-021-81411-4, PMID: 33500432 PMC7838397

[5] UNAIDS. Country factsheets –Brazil (2023) Available online at: https://www.unaids.org/en/regionscountries/countries/brazil

[6] Bastos FI Bastos LS Coutinho C Toledo L Mota JC Velasco-de-Castro CA. Divas research, G. HIV, HCV, HBV, and syphilis among transgender women from Brazil: assessing different methods to adjust infection rates of a hard-to-reach, sparse population. Medicine. (2018) 97:S16–24. doi: 10.1097/MD.0000000000009447 29794601 PMC5991532

[7] Jalil EM Torres TS Pereira CC d A Farias A JDU B Lacerda M. High rates of sexualized drug use or chemsex among Brazilian transgender women and young sexual and gender minorities. Int J Environ Res Public Health. (2022) 19:1704. doi: 10.3390/ijerph19031704 35162728 PMC8835457

[8] Teixeira SL Jalil CM Jalil EM Nazer SC Silva S Veloso VG. Evidence of an untamed HIV epidemic among MSM and TGW in Rio de Janeiro, Brazil: a 2018 to 2020 cross-sectional study using recent infection testing. J Int AIDS Soc. (2021) 24:e25743. doi: 10.1002/jia2.25743, PMID: 34132470 PMC8207443

[9] Winter S Diamond M Green J Karasic D Reed T Whittle S. Transgender people: health at the margins of society. Lancet. (2016) 388:390–400. doi: 10.1016/S0140-6736(16)00683-8, PMID: 27323925

[10] Brookfield S Dean J Forrest C Jones J Fitzgerald L. Barriers to accessing sexual health services for transgender and male sex workers: a systematic qualitative meta-summary. AIDS Behav. (2020) 24:682–96. doi: 10.1007/s10461-019-02453-4, PMID: 30868447

[11] Benson C Wang X Dunn KJ Li N Mesana L Lai J. Antiretroviral adherence, drug resistance, and the impact of social determinants of health in HIV-1 patients in the US. AIDS Behav. (2020) 24:3562–73. doi: 10.1007/s10461-020-02937-8, PMID: 32488554

[12] Justiça, CNd. Goiás se mantém como importante polo do tráfico internacional de mulheres [Internet]. (2015) Available online at: https://www.jusbrasil.com.br/noticias/goias-se-mantem-como-importante-polo-do-trafico-internacional-de-mulheres/181710455

[13] Baral SD Poteat T Stromdahl S Wirtz AL Guadamuz TE Beyrer C. Worldwide burden of HIV in transgender women: a systematic review and meta-analysis. Lancet Infect Dis. (2013) 13:214–22. doi: 10.1016/S1473-3099(12)70315-8, PMID: 23260128

[14] Heckathorn DD. Snowball versus respondent-driven sampling. Sociol Methodol. (2011) 41:355–66. doi: 10.1111/j.1467-9531.2011.01244.x, PMID: 22228916 PMC3250988

[15] Cardoso LPV Queiroz BB Stefani MMA. HIV-1 pol phylogenetic diversity and antiretroviral resistance mutations in treatmentnaive patients from central West Brazil. J Clin Virol. (2009) 46:134–9. doi: 10.1016/j.jcv.2009.07.009 19682948

[16] Delatorre E Silva-de-Jesus C Couto-Fernandez JC Pilotto JH Morgado MG. High HIV-1 diversity and prevalence of transmitted drug resistance among antiretroviral-naive HIV-infected pregnant women from Rio de Janeiro, Brazil. AIDS Res Hum Retrovir. (2017) 33:68–73. doi: 10.1089/AID.2016.0159, PMID: 27392995

[17] Tamura K Stecher G Kumar S. MEGA11: molecular evolutionary genetics analysis version 11. Mol Biol Evol. (2021) 38:3022–7. doi: 10.1093/molbev/msab120, PMID: 33892491 PMC8233496

[18] Altschul SF Gish W Miller W Myers EW Lipman DJ. Basic local alignment search tool. J Mol Biol. (1990) 215:403–10. doi: 10.1016/S0022-2836(05)80360-2, PMID: 2231712

[19] West BJ Massari GF Culbreth G Failla R Bologna M Dunbar RIM. Relating size and functionality in human social networks through complexity. Proc Natl Acad Sci USA. (2020) 117:18355–8. doi: 10.1073/pnas.2006875117, PMID: 32690712 PMC7414177

[20] Damacena GN Szwarcwald CL Souza Júnior PRB Ferreira Júnior OC Almeida WS Pascom ARP. Application of the respondent-driven sampling methodology in a biological and behavioral surveillance survey among female sex workers, Brazil, 2016. Rev Bras Epidemiol. (2019) 22:1–13. doi: 10.1590/1980-549720190002.supl.1 31576978

[21] Hosmer DW Lemeshow S. Applied logistic regression. 2nd ed. New York:John Wiley & Sons (2000).

[22] Borgatt SP Everett MG Johnson JC. Analyzing social networks. 2nd ed. London: S. Publicações (2018).

[23] Cesar GA Do Lago BV Ortiz Tanaka TS Zanini PB Bandeira LM MAM P. Differences in risky sexual behaviors and HIV prevalence between men who have sex with men and transgender women in the Midwest Brazil. PLOS Glob Public Health. (2024) 4:e0003061. doi: 10.1371/journal.pgph.0003061 38709753 PMC11073717

[24] Soares F MacCarthy S Magno L da Silva LAV Amorim L Nunn A. Factors associated with PrEP refusal among transgender women in northeastern Brazil. AIDS Behav. (2019) 23:2710–8. doi: 10.1007/s10461-019-02501-z, PMID: 30972620 PMC9982655

[25] Leite BO de Medeiros DS Magno L Bastos FI Coutinho C de Brito AM. Association between gender-based discrimination and medical visits and HIV testing in a large sample of transgender women in Northeast Brazil. Int J Equity Health. (2021) 20:199. doi: 10.1186/s12939-021-01541-z, PMID: 34488781 PMC8422640

[26] Amarante ICJ Lippman SA Sevelius JM Saggese GSR da Silva AAM Veras MASM. Anticipated stigma and social barriers to communication between transgender women newly diagnosed with HIV and health care providers: a mediation analysis. LGBT Health. (2024) 11:229–38. doi: 10.1089/lgbt.2023.0041, PMID: 37910864 PMC11001954

[27] Deutsch MB Glidden DV Sevelius J Keatley J McMahan V Guanira J. HIV pre-exposure prophylaxis in transgender women: a subgroup analysis of the iPrEx trial. Lancet HIV. (2015) 2:e512–9. doi: 10.1016/S2352-3018(15)00206-4, PMID: 26614965 PMC5111857

[28] Logie CH Lacombe-Duncan A Wang Y Jones N Levermore K Neil A. Prevalence and correlates of HIV infection and HIV testing among transgender women in Jamaica. AIDS Patient Care STDs. (2016) 30:416–24. doi: 10.1089/apc.2016.0145, PMID: 27610463

[29] Stahlman S Liestman B Ketende S Kouanda S Ky-Zerbo O Lougue M. Characterizing the HIV risks and potential pathways to HIV infection among transgender women in cote d'Ivoire, Togo and Burkina Faso. J Int AIDS Soc. (2016) 19:20774. doi: 10.7448/IAS.19.3.20774, PMID: 27431465 PMC4949310

[30] Rocha A Barros C Generoso IP Bastos FI Veras MA. HIV continuum of care among trans women and travestis living in Sao Paulo, Brazil. Rev Saude Publica. (2020) 54:118. doi: 10.11606/s1518-8787.2020054002374, PMID: 33237173 PMC7664846

[31] Grinsztejn B Jalil EM Monteiro L Velasque L Moreira RI Garcia AC. Unveiling of HIV dynamics among transgender women: a respondent-driven sampling study in Rio de Janeiro, Brazil. Lancet HIV. (2017) 4:e169–76. doi: 10.1016/S2352-3018(17)30015-2, PMID: 28188030 PMC5411266

[32] BRAZIL. Boletim Epidemiológico - HIV e Aids 2024. [Internet]. (2024). Available online at: https://www.gov.br/aids/pt-br/central-de-conteudo/boletins-epidemiologicos/2024/boletim_hiv_aids_2024e.pdf

[33] Stutterheim SE van Dijk M Wang H Jonas KJ. The worldwide burden of HIV in transgender individuals: an updated systematic review and meta-analysis. PLoS One. (2021) 16:e0260063. doi: 10.1371/journal.pone.0260063, PMID: 34851961 PMC8635361

[34] Becasen JS Denard CL Mullins MM Higa DH Sipe TA. Estimating the prevalence of HIV and sexual behaviors among the US transgender population: a systematic review and meta-analysis, 2006-2017. Am J Public Health. (2019) 109:e1–8. doi: 10.2105/AJPH.2018.304727, PMID: 30496000 PMC6301428

[35] Cardoso SW Torres TS Santini-Oliveira M Marins LM Veloso VG Grinsztejn B. Aging with HIV: a practical review. Braz J Infect Dis. (2013) 17:464–79. doi: 10.1016/j.bjid.2012.11.007, PMID: 23711587 PMC9428066

[36] Simela SR Kelepile M Sebobi TI. Spatial analysis and associated risk factors of HIV prevalence in Botswana: insights from the 2021 Botswana AIDS impact survey (BAIS V). BMC Infect Dis. (2025) 25:69. doi: 10.1186/s12879-025-10464-x, PMID: 39815215 PMC11736943

[37] Konda KA Torres TS Mariño G Ramos A Moreira RI Leite IC. Factors associated with long-term HIV pre-exposure prophylaxis engagement and adherence among transgender women in Brazil, Mexico and Peru: results from the ImPrEP study. J Int AIDS Soc. (2022) 25:e25974. doi: 10.1002/jia2.25974, PMID: 36225148 PMC9557020

[38] Kcomt L Evans-Polce RJ Boyd CJ McCabe SE. Association of transphobic discrimination and alcohol misuse among transgender adults: results from the U.S. transgender survey. Drug Alcohol Depend. (2020) 215:108223. doi: 10.1016/j.drugalcdep.2020.108223, PMID: 32777693 PMC7502497

[39] Sausa LA Keatley J Operario D. Perceived risks and benefits of sex work among transgender women of color in San Francisco. Arch Sex Behav. (2007) 36:768–77. doi: 10.1007/s10508-007-9210-3, PMID: 17674180

[40] Dolengevich-Segal H Rodríguez-Salgado B Bellesteros-López J Molina-Prado R. Chemsex. An emergent phenomenon. Adicciones. (2017) 29:207–9. doi: 10.20882/adicciones.894, PMID: 28492961

[41] Íncera-Fernández D Riquelme AR Sánchez-Ocaña A Montesinos F Gámez-Guadix M. A systematic review of intervention strategies aimed at chemsex users. Int J Drug Policy. (2025) 140:104795. doi: 10.1016/j.drugpo.2025.104795, PMID: 40334305

[42] Gräf T Bello G Andrade P Arantes I Pereira JM da Silva ABP. HIV-1 molecular diversity in Brazil unveiled by 10 years of sampling by the national genotyping network. Sci Rep. (2021) 11:15842. doi: 10.1038/s41598-021-94542-5, PMID: 34349153 PMC8338987

[43] Silva ÁMDCE Reis MNDG Marinho TA de Freitas NR Teles SA de Matos MAD. Epidemiological and molecular characteristics of HIV-1 infection in a sample of men who have sex with men in Brazil: phylogeography of major subtype B and F1 transmission clusters. Front Microbiol. (2020) 11:589937. doi: 10.3389/fmicb.2020.589937, PMID: 33329467 PMC7732656

[44] Tanaka TSO Leite TF Freitas SZ Cesar GA de Rezende GR Lindenberg ASC. HIV-1 molecular epidemiology, transmission clusters and transmitted drug resistance mutations in Central Brazil. Front Microbiol. (2019) 10:20. doi: 10.3389/fmicb.2019.00020, PMID: 30804893 PMC6371026

[45] Cardoso LP Pereira GA Viegas AA Schmaltz LE Stefani MM. HIV-1 primary and secondary antiretroviral drug resistance and genetic diversity among pregnant women from Central Brazil. J Med Virol. (2010) 82:351–7. doi: 10.1002/jmv.21722, PMID: 20087934

