# Supplementary material for: Epidemiological dynamics and molecular characteristics of HIV-1 among transgender women in the Central-West region of Brazil
Source: Front Public Health. 2025 Nov 5;13:1679535. doi: 10.3389/fpubh.2025.1679535 (PMC12626951; doi:10.3389/fpubh.2025.1679535)
Supplement: Supplementary file 1 [file Table_1.DOCX]

Supplementary Material

**Table S1. Characteristics of participants selected to be the seed of the recruitment through RDS method, Goiás, Brazil, 2018-2019**

| **Seeds ID** | **Age (years)** | **HIV status** | **Schooling (years)** | **Monthly income (BRL)^a^** | **Skin color (self-reported)** | **Profession** | | **Waves (N)** | **Recruits (N)** |
| --- | --- | --- | --- | --- | --- | --- | --- | --- | --- |
| XG-01 | 59 | Pos. | 12 | 3,000 | Black | Sex worker | 11 | | 141 |
| XG-02 | 39 | Pos. | 12 | 1,200 | Brown | Social educator | 22 | | 58 |
| XG-24 | 24 | Neg. | 11 | 1,800 | Brown | Sex worker | 3 | | 5 |
| XG-32 | 34 | Neg. | 18 | 3,500 | Brown | Teacher | 1 | | 2 |
| XG-50 | 23 | Neg. | 12 | 1,800 | Brown | Sex worker | 13 | | 74 |
| XJ-1 | 34 | Neg. | 10 | 3,000 | Brown | Hairdresser | 14 | | 80 |
| XI-1 | 33 | Pos. | 13 | 1,200 | Indigenous | Activist | 9 | | 53 |
| XI-51 | 35 | Neg. | 16 | 5,000 | White | Sex worker | 7 | | 19 |

ID: identification; BRL: Brazilian currency (*reais*); N: total number.

^a^BRL 3,91 was equivalent to USD 1 during the study.

**Table S2. Data of the detected phylogenetic clusters according to the HIV-1**

**subtype verified among transgender women in Goiás, Brazil, 2018-2019**

| **Cluster** | **Subtype** | **aLTR** | **Sequences** | **Genetic Distance (%)** | **Final status** |
| --- | --- | --- | --- | --- | --- |
| 1 | B | 1 | XG048 | 14.7 | Not confirmed |
|  |  |  | XG263 |  |  |
| 2 | B | 1 | XJ061 | 1.2 |  |
|  |  |  | XG224 |  | Confirmed |
|  |  |  | XJ026 |  |  |
| 3 | B | 0.99 | XI019 | 6.0 | Not confirmed |
|  |  |  | XI020 |  |  |
| 4 | B | 1 | XI001 | 1.6 | Confirmed |
|  |  |  | XI014 |  |  |
| 5 | B | 0.97 | XG119 | 8.6 | Not confirmed |
|  |  |  | XG063 |  |  |
| 6 | C | 1 | XG124 | 2.8 |  |
|  |  |  | XG070 |  | Confirmed |
|  |  |  | XI017 |  |  |
| 7 | C | 1 | XI067 | 2.5 | Confirmed |
|  |  |  | XI052 |  |  |
| 8 | C | 1 | XG067 | 3.2 | Confirmed |
|  |  |  | XG222 |  |  |
| 9 | F1 | 1 | XI050 | 2.9 |  |
|  |  |  | XI057 |  | Confirmed |
|  |  |  | XI061 |  |  |
| 10 | F1 | 1 | XJ012 | 2.3 | Confirmed |
|  |  |  | XG144 |  |  |
| 11 | F1 | 1 | XG139 | 1.8 |  |
|  |  |  | XG272 |  |  |
|  |  |  | XG138 |  | Confirmed |
|  |  |  | XG011 |  |  |
| 12 | BF | - | XG122 | 2.3 | Confirmed |
|  |  |  | XG088 |  |  |
| 13 | BF | - | XG082 | 1.5 | Confirmed |
|  |  |  | XG171 |  |  |

aLTR: Alternative likelihood

**Table S3. Characteristics of the transgender women (N = 34) presenting with drug resistance mutations. Goiás, Brazil, 2018-2019**

| **Identification** | **HIV-1 Subtype** | **Reported ARV use** | **PI** | **NRTI** | **NNRTI** |  |
| --- | --- | --- | --- | --- | --- | --- |
| 2018BRGO_XG022 | B | yes |  | T215S |  |  |
| 2018BRGO_XG025 | B | no |  | F77L | V106I |  |
| 2018BRGO_XG067 | C | no |  |  | K103N |  |
| 2018BRGO_XG070 | C | yes |  |  | V108I |  |
| 2018BRGO_XG076 | B | no |  | S68G | E138A |  |
| 2018BRGO_XG082 | FB | no |  |  | V179E |  |
| 2018BRGO_XG090 | F | no |  |  | K103N |  |
| 2018BRGO_XG130 | B | yes |  | M184V | K103N, V108I, P225H, Y318F |  |
| 2018BRGO_XG131 | B | yes |  | M184V, T215A | K101P, K103N, V106I |  |
| 2018BRGO_XG138 | F | yes |  |  | V106I |  |
| 2018BRGO_XG141 | F | yes |  |  | V179T |  |
| 2018BRGO_XG144 | F | no |  | K65N |  |  |
| 2018BRGO_XG146 | B | no |  | K70N |  |  |
| 2018BRGO_XG181 | B | no |  |  | V179D |  |
| 2018BRGO_XG202 | B | ND |  | S68G |  |  |
| 2018BRGO_XG217 | B | no |  |  | V106I |  |
| 2018BRGO_XG222 | C | yes |  |  | K103N |  |
| 2019BRGO_XG225 | B | yes |  | S68G,V75M, M184V |  |  |
| 2019BRGO_XG263 | B | yes |  | S68G | K103N |  |
| 2019BRGO_XG272 | F | yes |  |  | V179D |  |
| 2018BRGO_XI014 | B | yes |  | S68G |  |  |
| 2018BRGO_XI017 | C | no |  | S68G, M184I | V108I, M230I |  |
| 2018BRGO_XI019 | B | yes | K20T, M46I, N88S | K70G, M184V |  |  |
|  |  |  |  |  |  |  |
| 2018BRGO_XI029 | B | no | Q58E | L74I, Y115F, M184V | V106IM, V179D, G190A, F227L |  |
| 2018BRGO_XI035 | BF | yes | M46MI |  |  |  |
| 2018BRGO_XI046 | B | no |  | M184I | M230I |  |
| 2018BRGO_XI055 | F | no | G48E, N83D |  | G190K |  |
| 2018BRGO_XI061 | F | no | V82A | S68G |  |  |
| 2018BRGO_XI070 | FC | no |  | S68G |  |  |
| 2018BRGO_XI072 | B | no |  | S68G |  |  |
| 2019BRGO_XJ019 | CRF39 | no | L10F |  | A98G |  |
| 2019BRGO_XJ028 | B | yes |  | S68G |  |  |
| 2019BRGO_XJ058 | B | yes |  |  | V179D |  |
| 2019BRGO_XJ061 | B | no |  |  | M230I |  |

ARV: antiretroviral; NNRTI: Non-Nucleoside ReverseTranscriptase Inhibitor; NRTI: Nucleoside Reverse

Transcriptase Inhibitor; PI: Protease Inhibitor.

**
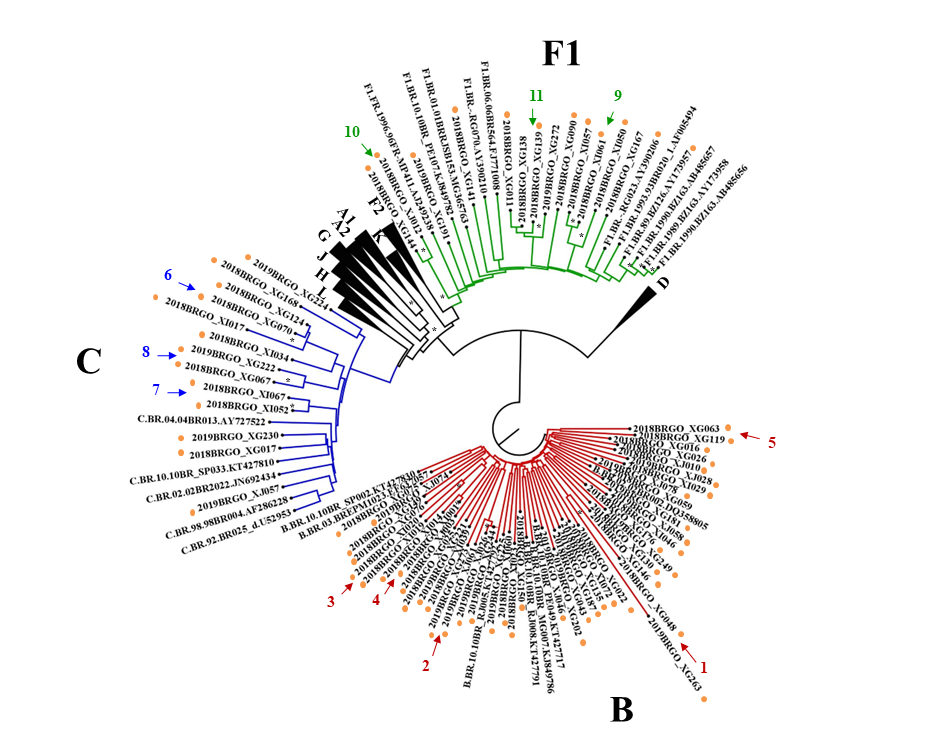

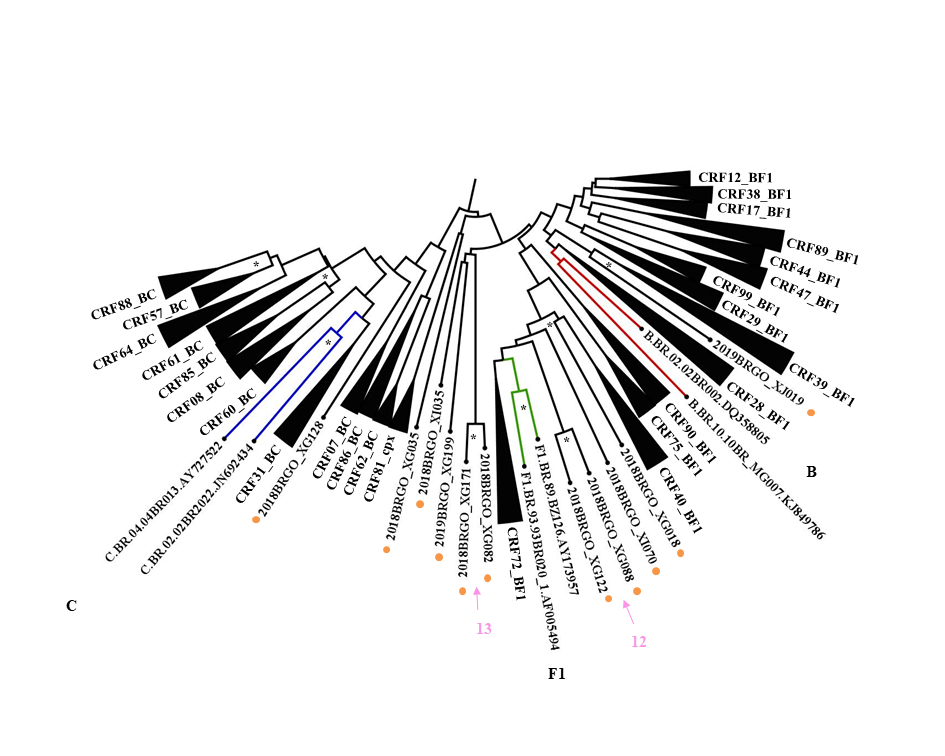
**

(B)

(A)

**Figure S1.** MJ phylogenetic tree of 82 HIV-1 PR/RT sequences from transgender women in Goiás. The analyzed PR/RT alignment covered a fragment of 1300 bp, corresponding to nucleotides 2253 to 3554 relative to HXB2 genome. The sequences obtained at the present study were identified by named according to the collected year and place (XG-Goiania, XI-Itumbiara and XJ-Jataí, Goiás, Brazil) and identified by orange circles. The clusters were colored by HIV-1 subtype B (red), C (blue), F1 (green). Clusters presenting only reference sequences were collapsed. Possible transmission clusters were numbered. Five short sequences were not included in the final phylogeny. Cluster depicting bootstrap >90 was represented with an asterisk. (A) Pure HIV-1 subtypes and (B) Recombinant sequences.
